# Fishery-based adaption to climate change: the case of migratory species flathead grey mullet (*Mugil cephalus* L.) in Taiwan Strait, Northwestern Pacific

**DOI:** 10.7717/peerj.15788

**Published:** 2023-08-30

**Authors:** Ming An Lee, Sandipan Mondal, Sheng-Yuan Teng, Manh-Linh Nguyen, Platinasoka Lin, Jun-Hong Wu, Biraj Kanti Mondal

**Affiliations:** 1 Center of Excellence for Oceans, National Taiwan Ocean University, Keelung, Zhongzheng District, Taiwan; 2 Environmental Biology & Fishery Science, National Taiwan Ocean University, Keelung, Zhongzheng District, Taiwan; 3 Institute of Ecology and Evolutionary Biology, National Taiwan University, Taipei City, Taiwan; 4 Taiwan Ocean Conservation and Fishery Sustainability Foundation, Taipei, Taiwan; 5 Department of Geography, Netaji Subhas Open University, Kolkata, West Bengal, India

**Keywords:** Flathead grey mullet, *Mugil cephalus*, Fisheries sustainability, Taiwan strait, Climate indexes

## Abstract

The flathead gray mullet (*Mugil cephalus L*.) is a cosmopolitan fish that lives in warm and temperate zones over 42°N–42°S. It is a key fish species for industrial fishing off coastal Taiwan. Gray mullets enter the coastal waters of the southeastern Taiwan Strait (22°N–25°N) to spawn in winter and feed in the coastal and tidal waters of China (25°N–30°N). From 1986 to 2010, the annual catch of gray mullet decreased substantially and remained low. Although the Pacific Decadal Oscillation and El Niño–Southern Oscillation are recognized to affect gray mullet migration, the increase in sea surface temperature may be the main cause of the aforementioned decrease. We explored how weather changes affect fishing conditions and patterns at the gray mullet fishing grounds in Taiwan’s coastal areas. Because of the decrease in gray mullet catches, the most common method for catching gray mullet in Taiwan’s coastal areas between 1990 and 2010 was the use of drift or trawl nets instead of two-boat purse-seiner fleets. Since 2012, purse-seiner fleets have become the most common method for catching gray mullet. This trend indicates that the local fishing industry is adapting to changing environmental conditions.

## Introduction

The flathead gray mullet (*Mugil cephalus L*.) inhabits the tropical and temperate regions between 42°N and 42°S (Thomson, 1963), and it is a highly valuable species for Taiwan’s coastal fisheries. Consequently, the locals call it the “Gray Gold.” Studies (Tung, 1981; Huang & Su, 1986; Shyu & Lee, 1986; Huang, 1989) have noted that gray mullet roe is suitable for consumption as a delicacy in Taiwan and Japan. According to Khemis, Hamza & Sadok (2019), the consumption of this species is beneficial to human health because of its high nutritional content. It can also be used to enhance the sediment quality in agricultural systems where multiple types of crops are present (Lupatsch, Katz & Angel, 2003).

**Figure 1:**
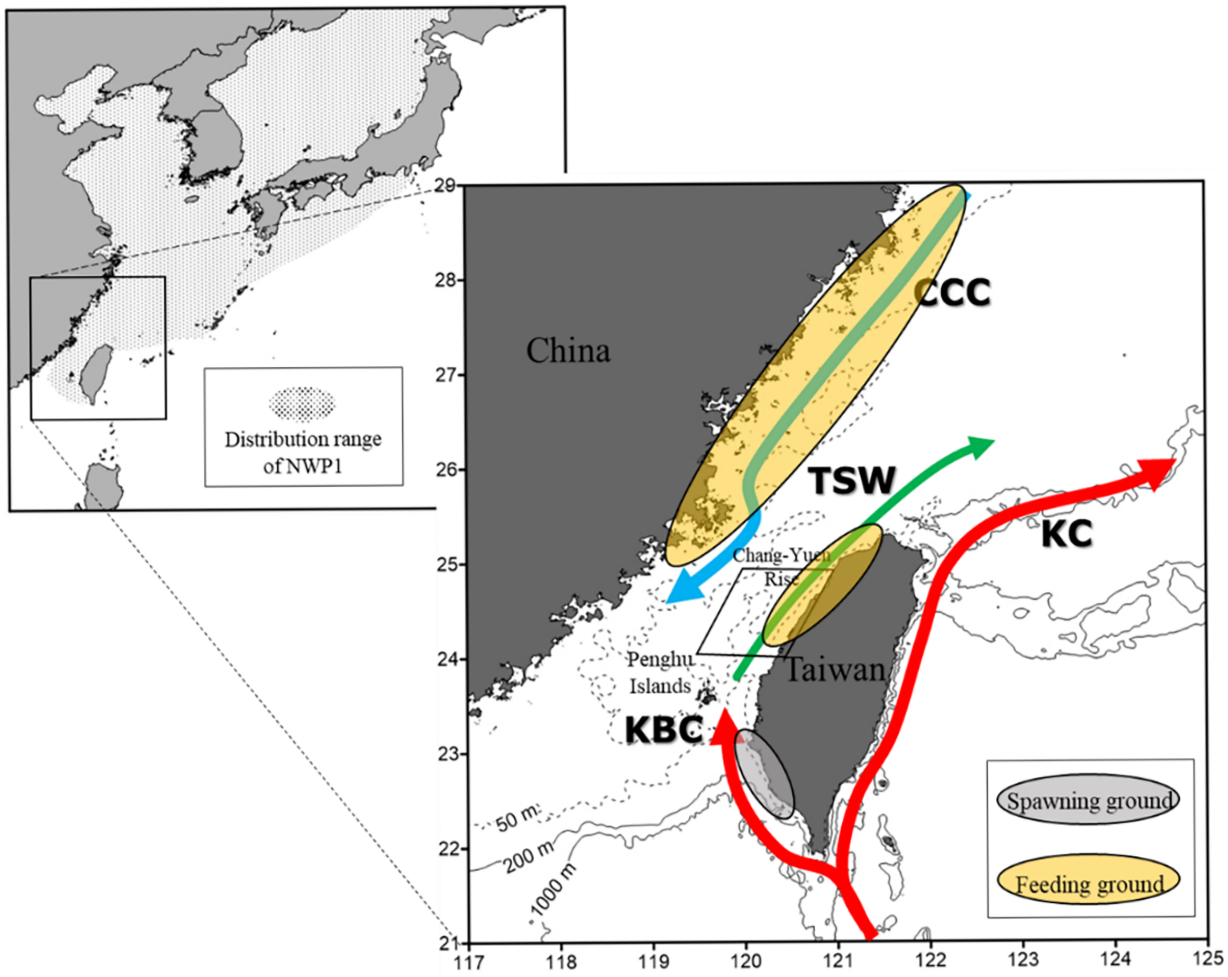
Grey mullet catch area around Taiwan’s coast during the study period.

The Flathead gray mullets caught in Taiwan reproduce and nurture their young in the coastal waters of Southwest Taiwan. Between 25°N and 30°N, the feeding ground of the gray mullet (both juvenile and adult) is located in the coastal and estuarine waters of mainland China (Fig. 1; Tung, 1981; Liu, 1986; Huang & Su, 1989). Because of the limited feeding ground in the coastal and estuarine waters of Northwest Taiwan, young gray mullets (>60 mm) are uncommon in these areas (Tung, 1981; Koga, 1958). In winter, gray mullets migrate from the coast of mainland China along the frigid China Coastal Current (CCC) to the coast of Central Taiwan (near Taichung) (Cheng et al., 2016). Figure 1 depicts the cold current moving south along the coast while the mild Southwest Taiwan branch of the Kuroshio Current moves north (Jan et al., 2002; Matsuno, Lee & Yanao, 2009; Hsu, Lee & Lu, 2021). Gray mullets congregate in large clusters at the front between cold and warm currents where the temperature changes (Chen & Su, 1986). Because of the presence of these clusters, gray mullet can be caught in the shallow waters off the west coast of Taiwan, particularly near Taoyuan and Fangliao (Shyu & Lee, 1986). Figure 1 demonstrates that the optimal area for catching gray mullet is a narrow 80-NM tract that is located between Anping and Fangliao and 1–4.5 NM from Taiwan’s coast. In this region, the bottom is sandy, the depth range is 10–30 m, the sea surface temperature (SST) range is 13–20.5 °C, and the salinity range is 34–34.8 parts per thousand (ppt) (Huang & Su, 1986; Huang, 1989; Kuo, 1986; Shyu & Lee, 1986; Tung, 1981).

According to Lan et al. (2014), the gray mullet fishing season runs from late November to early February. The period extending 10 days before and after the winter solstice is regarded as the optimal period for catching gray mullet (Huang & Su, 1986; Kuo, 1986; Tung, 1981). Gray mullet is typically caught using purse seines, drift gill nets, trap nets, and encircling nets (Huang & Su, 1986; Tung, 1981). Lan et al. (2014) examined the fishing techniques used to catch gray mullets during the period from 1967 to 2009, and they discovered a shift from using purse seines to using gillnets. In addition, they discovered that the gray mullet population in the Taiwan Strait declined from 2,503 × 10^3^ in 1982 to less than 300 × 10^3^ in 2009. Although the organization responsible for administering Taiwan’s fisheries could not explain the rapid decrease in this number in recent years, a possible explanation is that global climate change, which has caused Taiwan’s SST to increase by >1 °C in Huang & Su (1986) and Huang (1989) discovered a correlation between this decrease and October’s coldest month. Since 1986, fishing has become less economically viable, leading to a considerable decrease in annual fish catches. Since the 1980s, Taiwan has been catching a high number of gray mullets annually, and Lan et al. (2017) suggested that this trend contributed to the increased sensitivity of gray mullets to sudden alterations in their environment.

Taiwan’s gray mullet population has been negatively affected by overfishing and weather changes, resulting in a decrease in the total catch of this target species (Lan et al., 2014, 2017). According to Sustainable Development Goal (SDG) 14 of the United Nations (Cormier & Elliott, 2017), the oceans, seas, and marine resources must be protected and used responsibly, and the Taiwan Fisheries Agency has announced various policies and regulations that align with this goal (Ho, 2022; Mugagga & Nabaasa, 2016; Ju et al., 2020; Friess et al., 2019). Sustainable development is a concept emphasizing a balance between economic development, human development, and ecological protection. Sustainable use and ocean protection are two SDG 14 objectives (Griggs et al., 2017), and they entailing ending overfishing, preserving marine ecosystems, and minimizing the effects of ocean acidification and marine pollution (Barclay et al., 2020). An objective of sustainable fishing is to determine what proportions of the world’s fish populations have been overfished, fully exploited, or left untouched (Cormier & Elliott, 2017; Mugagga & Nabaasa, 2016; Ju et al., 2020). A fish stock that is partially or wholly depleted, whether by overfishing or otherwise, can no longer be considered sustainable (Kenny et al., 2018). Thus, the habitat preferences and ranges of gray mullet and other species must be comprehensively understood for their sustainability to be protected (Kumaran et al., 2012).

In the present study, we determined the cyclical characteristics associated with the commercial fishing of gray mullets in the western coastal waters of Taiwan and evaluated the meteorological factors that could have contributed to the recent decrease in gray mullet catches. The present study’s primary objective was to examine the effect of global warming and the El Niño phenomenon on the abundance of the migratory stock of gray mullet in Taiwan’s western coastal waters. A questionnaire was administered to examine the effects of extreme phenomena on the gray mullet fisheries in the study area and how they have adapted to climate change.

## Materials and Methods

### Data collection

#### Fishery data

**Figure 2:**
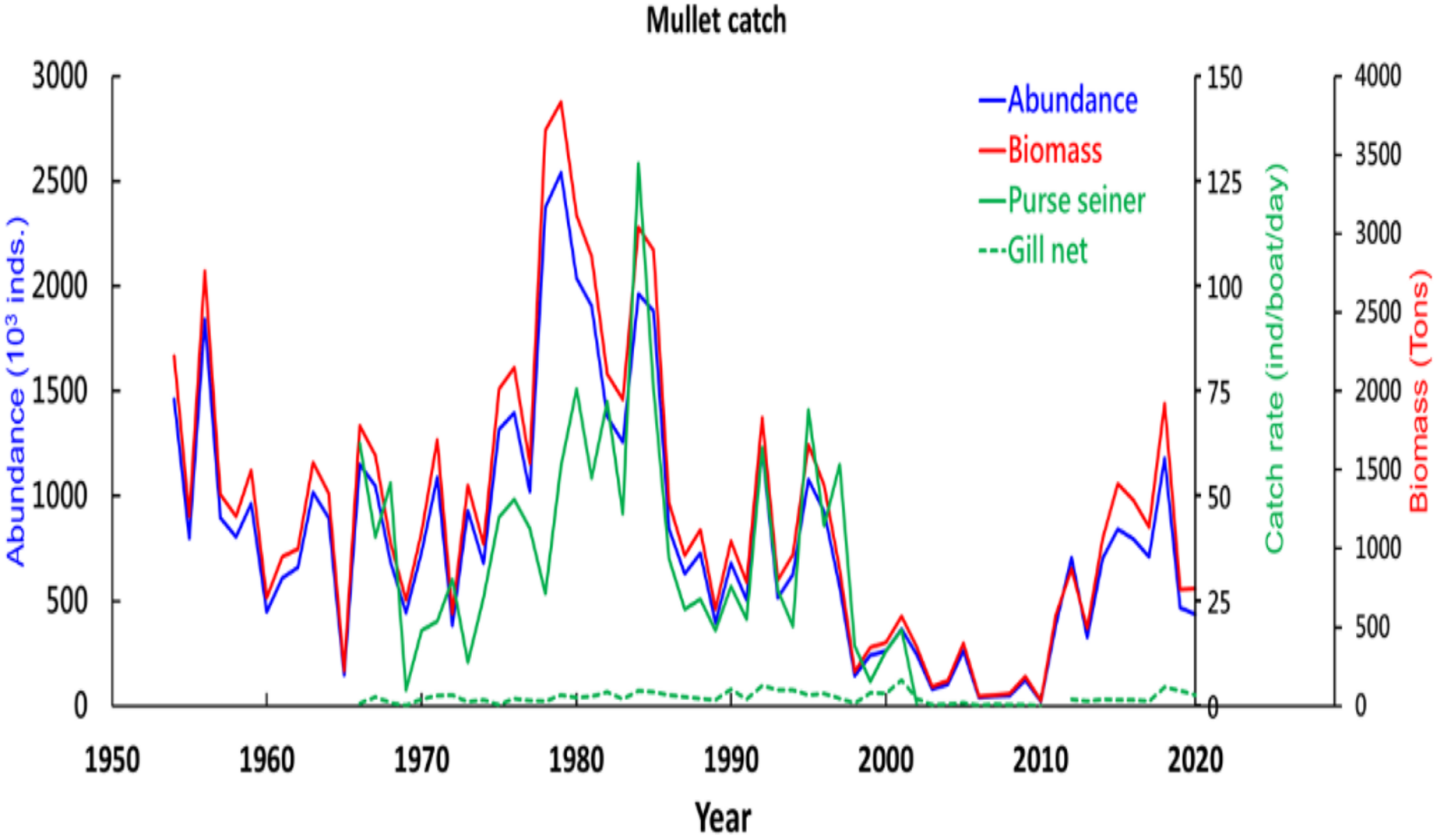
Grey mullet catch around Taiwan coast during the study period.

Gray mullet catch data from all Taiwanese fishery associations were obtained from the Kaohsiung Branch of the Fisheries Research Institute (Coastal and Offshore Resources Research Center of the Fisheries Research Institute). Figure 2 presents the dates and daily catches recorded during the fishing seasons from 1967 to 2020 and the annual catches from 1954 to 2020. The fishery data covered the area bounded by 21°N–29°N and 117°E–125°E (spatial resolution, 0.1°). The logbook data contained information on the year, month, latitude, longitude, weight of fish caught (measured in kilograms (kg)), fishing effort (measured in hours), total catch weight (dry or moist), fishing gear employed (primarily trawl nets, gill nets, or purse seines), and vessel identification number. However, information regarding fishing depth and apparatus soaking time was unavailable.

#### Climate data

The Central Weather Bureau collects climate data from five stations, namely the Penghu, Pengjiao, Tamshui, Fangliao, and Kaohsiung stations. From 1958 to 2018, the bureau collected daily atmospheric temperature, atmospheric pressure, wind velocity, and wind direction measurements. In addition, it collected data on the West Pacific Oscillation, Pacific Decadal Oscillation (PDO), North Pacific Gyre Oscillation (NPGO), Southern Oscillation Index, Arctic Oscillation Index, Oceanic Nio Index, and North Pacific Index.

Groups of mature gray mullets migrate south during winter from China’s coastal waters along the frigid North China Current (NCCC) to the eastern and central sections of the Taiwan Strait. We used the SST fluctuations at the Chang-Yuen Rise, Taiwan (Tzeng, Lan & Chan, 2012), to characterize changes in the NCCC. Simple Ocean Data Assimilation (version 2.2.4), which employed 20Crv2 winds and was the first assimilation run to be performed in more than a century, was used to obtain monthly SST data pertaining to the 1958–2018 period (Carton & Giese, 2008). Our ocean model’s numerical foundation was based on the Parallel Ocean Program (version 2.0.1).

### Modeling fisheries and environmental data

Without assumptions of stationarity (Ménard et al., 2007; Torrence & Compo, 1998), wavelet transformation analysis was performed on the basis of the convolution of a time series (*n* = 0, N − 1; with equal spacing) and a wavelet function (Ménard et al., 2007; Torrence & Compo, 1998). In this analysis, the Morlet wavelet function was employed. Before the wavelet analysis was conducted, we excluded the long-term linear trend because our focus was the interannual variation. On the basis of 1,000 bootstrap simulations that were treated as a first-order autoregressive process, the significance threshold of 5% was determined. We conducted cross-wavelet coherence and phase experiments to further investigate the causal connections between environmental variables and catch rates. Cross-wavelet coherence is cross-correlation normalized to the power of a single series (Grinsted, Moore & Jevrejeva, 2004). A wavelet transform generates edge artifacts because a wavelet lacks temporal confinement. Therefore, introducing a cone of impact where the effects at the margins cannot be ignored is advantageous (Grinsted, Moore & Jevrejeva, 2004; Ménard et al., 2007). However, when finite-duration time series are used, errors appear at the beginning and conclusion of the wavelet power spectrum. To address this problem, zeros are added to the end of the time series prior to the application of a wavelet transform, and these zeros are subsequently removed (Meyers, Kelly & O’Brien, 1993; Torrence & Compo, 1998). In the present investigation, wavelet analysis was primarily conducted to determine the relationship between climate and catch data.

### Perceptions of fisheries

We administered a questionnaire to 46 fishery stakeholders, including fishermen (*n* = 31), government personnel (*n* = 4), and researchers who specialized in fisheries research (*n* = 11), to understand their perspectives. The first section of our questionnaire collected basic participant information. The second section comprised five key questions regarding changes in the marine environment (Table 1). The first question centered on the economic effects of climate change in terms of variations in operating costs, fishing scale, primary catch, and overall benefits. The second and third questions pertained to the impact of environmental changes and fishing operations, respectively. Responses to all components of these three questions were scored on the basis of the level of agreement, which ranged from “*strongly disagree*” to “*strongly agree*.” The remaining two questions required open-ended responses.

**Table 1:** Main part of our questionnaire survey.

Main multiple choice question: Based on your own experience or observations, which grey mullet fishery–related elements have demonstrated variation over time?					
	Strongly agree	Agree	Not certain/not applicable	Disagree	Strongly disagree
1. Economic aspects					
Increasing operating costs					
Reduced operational scale					
Changes in main catch in terms of body size					
Decreasing fish production					
Declining overall benefit					
2. Environment and ecology					
Increasing seawater temperature					
Variations in oceanic currents					
Increasing frequency of extreme weather					
3. Fishing operations					
Variations in fishing grounds					
Variations in operating time during the fishing season					
Increased difficulty in operation of fishing vessels					
Variations in vessel sizes					
Short answer questions:					
4. What challenges are Taiwan’s grey mullet fisheries industry facing?					
5. In your opinion, what would be the best solution for resolving the aforementioned challenges?					
6. What do you think of sustainable development for grey mullet fisheries in Taiwan?					

## Results

### Catch variability of gray mullet

#### Catch variability over time

Two notable trends were noted in the variation in gray mullet catch in the Taiwan Strait from 1954 to 2020, namely an increase in gray mullet biomass between 1970 and 1980 followed by a rapid decrease after 1987 (Fig. 2). The catch rate was lowest in 2010 but increased subsequently. Throughout the study period, purse seines were the most commonly used fishing gear for catching gray mullet. The number of gray mullets caught peaked in 1980, (*n* = 3,000), with the corresponding catch biomass and catch rate approaching 4,000 t and 150 fish/boat/day, respectively. By contrast, fewer than 500 fish were caught in 2010, and the corresponding capture biomass and catch rate only approached 500 t and 5 fish/boat/day, respectively. Throughout the study period, purse seines were used more frequently relative to gill nets.

**Figure 3:**
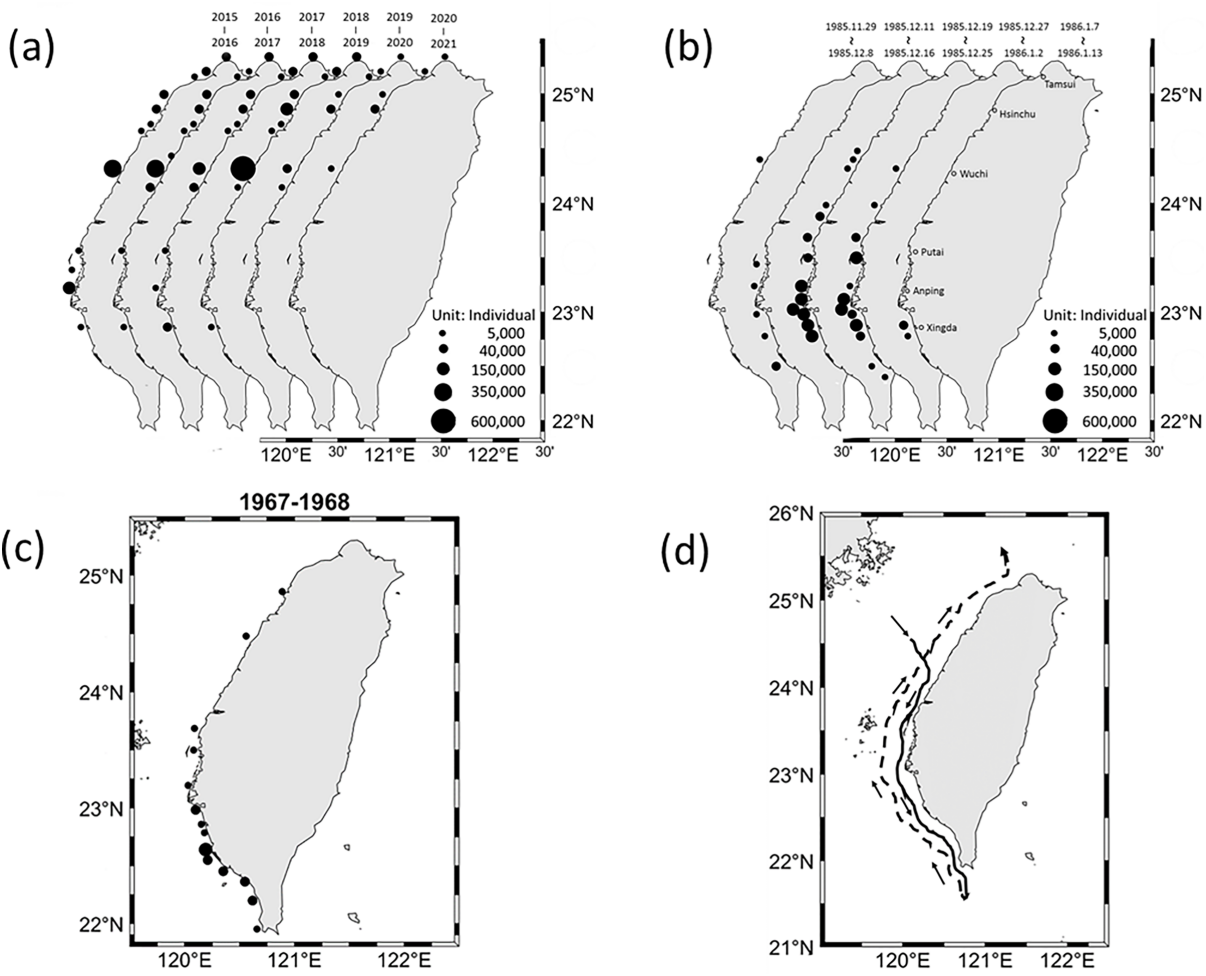
Grey mullet catch distribution in coastal waters of Taiwan. (A) 2015–2019 (data from Fisheries Agency, Council of Agriculture, Executive Yuan), (B) 1985–1986 (Lin, 1979), and (C) 1967–1968. (D) Grey mullet migration path in 1920 (Ōshima, 1922).

The gray mullets caught in the coastal waters of Taiwan were primarily caught between 22°N and 23°N during the period from 1967 to 1968 and between 23°N and 24°N in 1985 (Fig. 3). However, from 2015 onward, the distribution of gray mullets changed considerably. During the 2015–2020 period, gray mullet was primarily caught between 24°N and 25°N, particularly 24.3°N. The migration of gray mullet exhibited a notable pattern; specifically, they migrated southward between 1967 and 1968 but they migrated northward until 25°N after 2000.

#### Catch variability in relation to latitude

**Figure 4:**
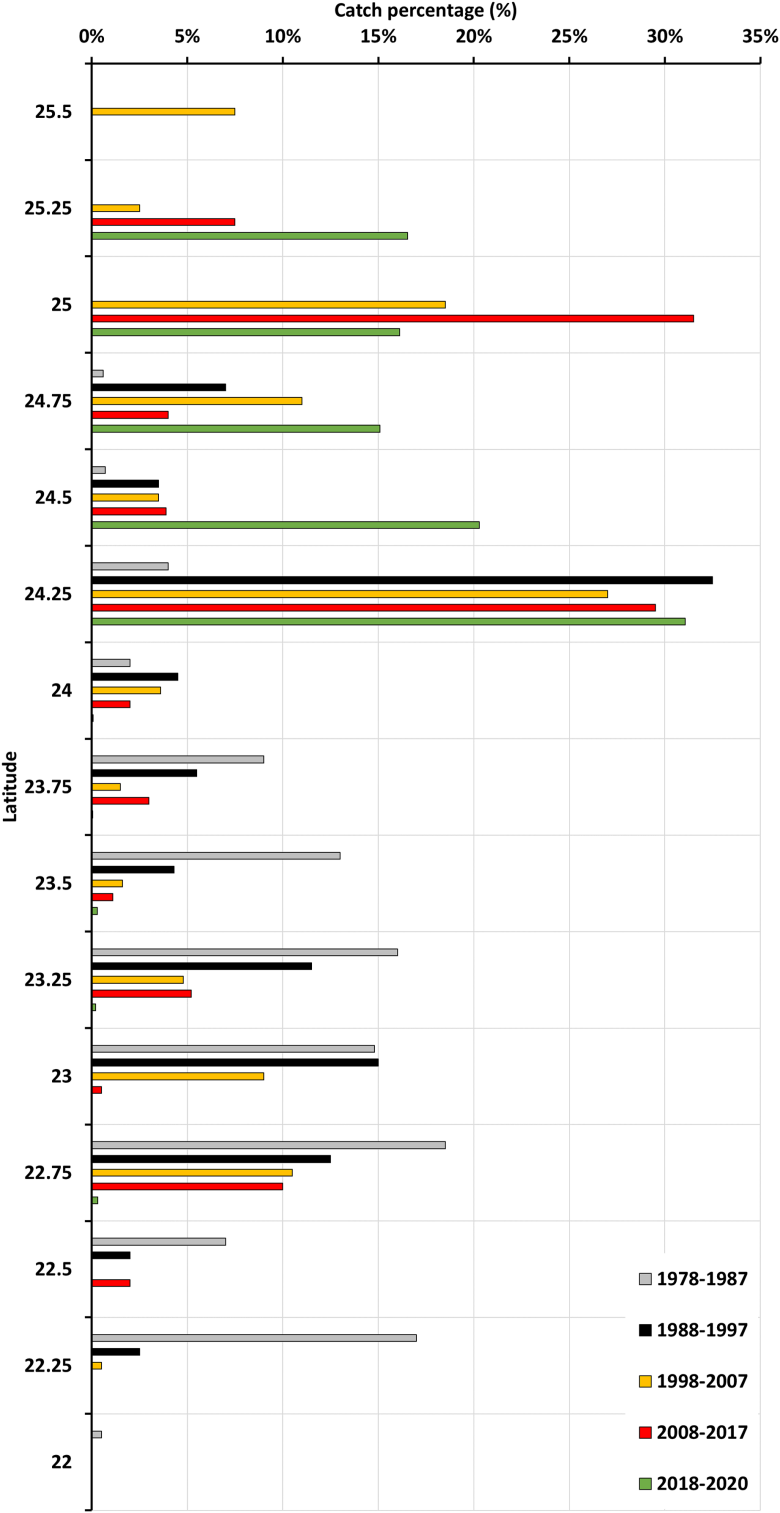
Latitudinal variation in gray mullet catches during 1978–2020 period.

The latitudinal variations in gray mullet catches from 1978 to 2020 exhibited a prominent latitudinal shift between 1978 and 1987 and between 2018 and 2020 (Fig. 4). During the 1978–1987 period, catches were mainly distributed between 22°N and 24°N. However, during the 1998–2007 period, catches were mainly distributed between 24°N and 24.5°N. Furthermore, during the 2017–2018 and 2018–2020 periods, catches were mainly distributed between 24.5°N and 25°N and between 24°N and 25.5°N, respectively. Overall, a latitudinal shift occurred between 22°N and 25.5°N in terms of the percentage of catches.

#### Catch variability in relation to type of fishing gear used

**Figure 5:**
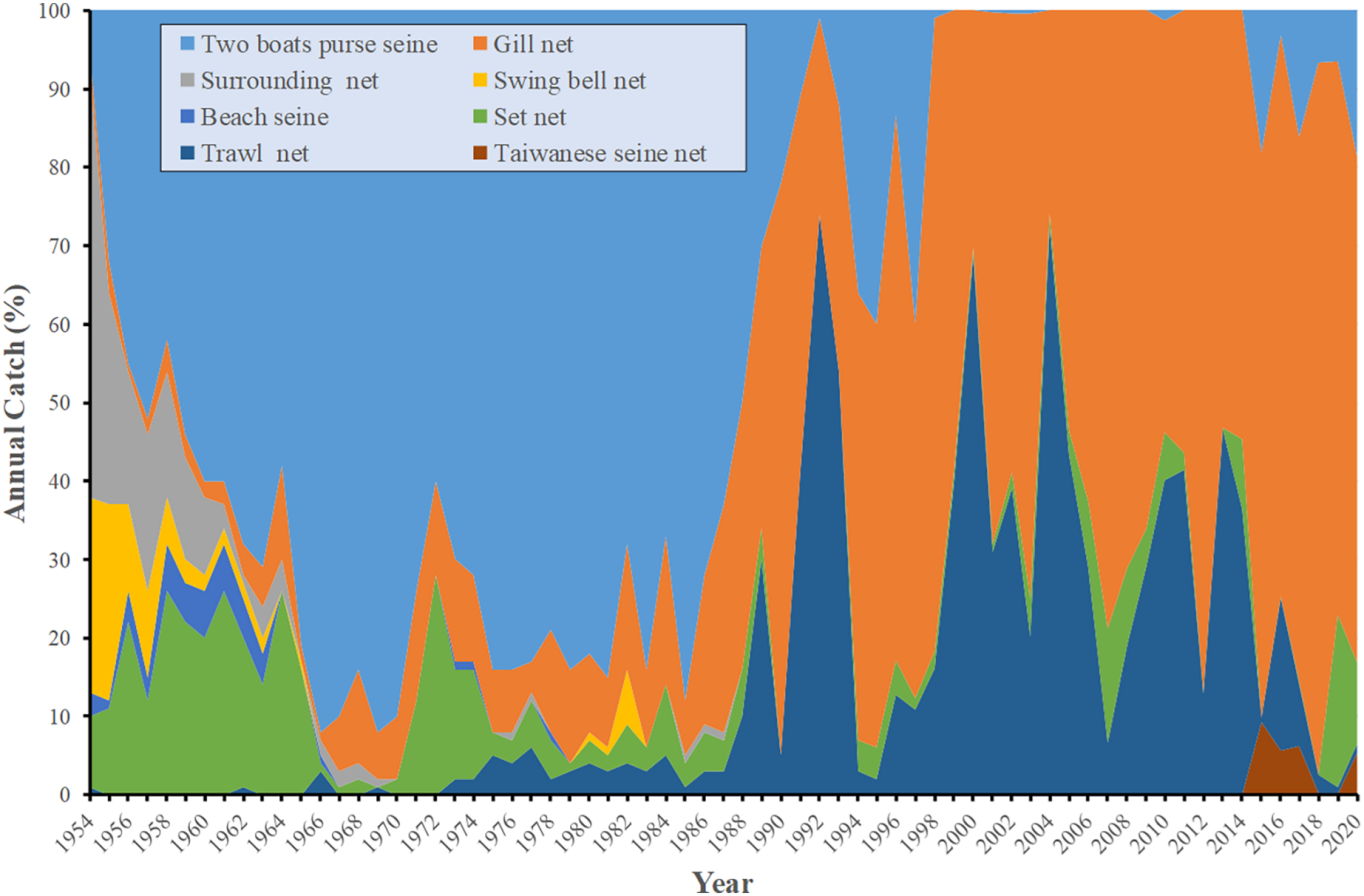
Catch variability in relation to fishing gear.

Between 1954 and 1960 (Fig. 5), the most commonly used fishing method was the use of two-boat purse seiners (*n* = 41), followed by the use of vessels with surrounding nets (*n* = 23). During the 1961–1970 and 1971–1980 periods, two-boat purse seiners were the most commonly used fishing vessels (1961–1970, *n* = 78; 1971–1980, *n* = 77). Between 1981 and 1990, the use of two-boat purse seiners (*n* = 62) and the use of gill nets (*n* = 25) were the most commonly employed fishing methods. Between 1991 and 2000, gill nets (*n* = 50) and trawl nets (*n* = 32) were more commonly used relative to two-boat purse seiners (*n* = 15). By the 2001–2010 period, two-boat purse seiners have mostly been phased out; during this period, most fishing vessels used gill nets (*n* = 61) or trawl nets (*n* = 33). Between 2011 and 2020, the most commonly used fishing gear (Table 2) was the gillnet.

**Table 2:** Grey mullet fishing boats by period.

Fishing gear	Two boat purse seine	Gill net	Surrounding net	Swing bell net	Beach net	Set net	Trawl net	Taiwanese seine net	Mean ± SD	
Year									Catch (tons)	PDO
1954–1960	41	2.43	23.29	11.71	4	17.43	0.14	0	1,559.9 ± 700.5	−0.4 ± 1.12
1961–1970	78.6	6	1.9	0.7	1.6	10.7	0.5	0	1,125.6 ± 465.4	−0.5 ± 0.32
1971–1980	77.2	10.5	0.2	0.1	0.3	8.9	2.8	0	2,102.5 ± 1,099	−0.49 ± 0.68
1981–1990	62.9	25.2	0.3	0.8	0	4.1	60.7	0	1,788.3 ± 905.8	0.32 ± 0.74
1991–2000	15.42	50.51	0	0	0	1.82	32.25	0	930.3 ± 547.4	−0.06 ± 1.05
2001–2010	0.22	61.11	0	0	0	5.67	33	0	205.9 ± 180.7	−0.64 ± 0.59
2011–2020	6.93	68.95	0	0	0	4.44	16.97	0	1,027 ± 435.6	−0.43 ± 0.99

### Relationship between gray mullet catch and environmental conditions

#### Pearson correlation analysis

**Figure 6:**
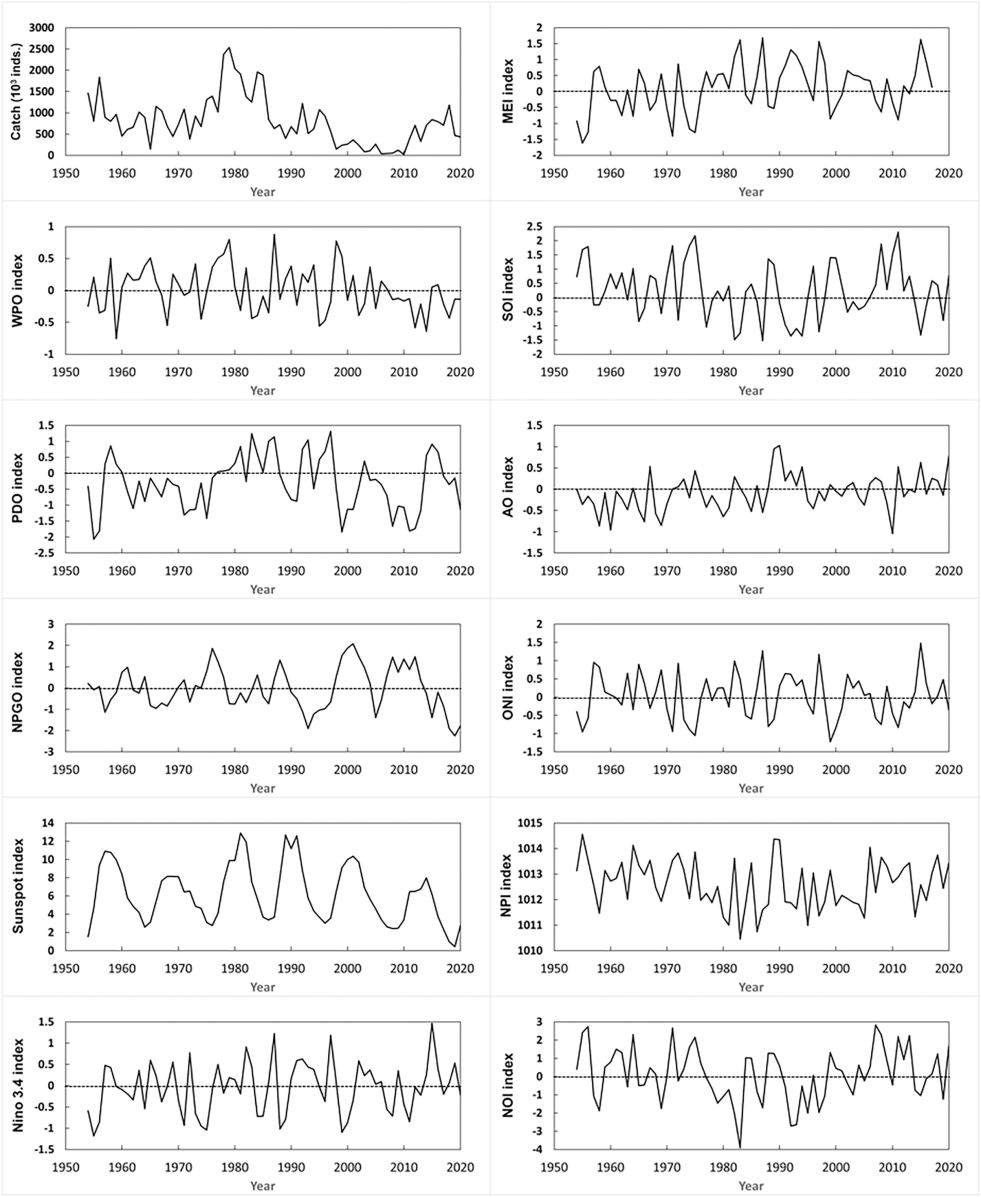
Annual changes in oceanographic oscillation indexes.

The annual variations of several oceanographic oscillation indices throughout the study period were examined (Fig. 6). In addition, Table 3 lists the *r* values for annual biomass and abundance of gray mullet in relation to environmental variables since 1912. None of the oceanographic indices exhibited a dominant trend during the study period. However, various oceanographic indices affected gray mullet catches during various seasons.

**Table 3:** Pearson correlation coefficients of the annual mullet biomass (A) and abundance (B) related to the environmental variables since 1912.

(A) Factors	Year	Autumn	Winter
WPO	−0.03	−0.13	−0.33
PDO	0.29	0.38	0.16
NPGO	−0.29	−0.24	−0.24
Sunspot	0.20	0.19	0.19
Nino 3.4	0.00	−0.01	0.03
MEI	−0.02	0.04	0.76
SOI	−0.09	−0.14	−0.07
AO	0.00	0.03	0.03
ONI	0.05	0.04	0.02
NPI	−0.10	−0.15	0.02
NOI	−0.16	−0.15	−0.12
SST anomaly in C-Y rise	−0.48	−0.46	−0.46
SST anomaly in coastal water of China	−0.43	−0.42	−0.37
Wind speed in C-Y rise	0.41	0.34	0.34
Wind speed in coastal water of China	0.42	0.33	0.33

(B) Factors	Year	Autumn	Winter
WPO	−0.04	−0.12	−0.34
PDO	0.28	0.37	0.15
NPGO	−0.28	−0.23	−0.24
Sunspot	0.21	0.20	0.20
Nino 3.4	0.00	−0.01	0.02
MEI	−0.02	0.03	−0.01
SOI	−0.08	−0.14	−0.07
AO	−0.01	0.03	0.03
ONI	0.05	0.04	0.02
NPI	−0.10	−0.15	0.02
NOI	−0.16	−0.15	−0.12
SST anomaly in C-Y rise	−0.49	−0.47	−0.47
SST anomaly in coastal water of China	−0.44	−0.43	−0.37
Wind speed in C-Y rise	0.42	0.34	0.34
Wind speed in coastal water of China	0.42	0.34	0.34

The annual biomass of gray mullet since 1912 (Table 3) exhibited the strongest negative correlation with the SST anomaly at the Chang-Yuen Rise, followed by the SST anomaly in the littoral waters of China; the biomass of gray mullets during autumn and winter exhibited comparable trends. The strongest positive correlation between wind speed and the annual biomass of gray mullets was identified in the coastal waters of China, followed by the Chang-Yuen Rise, where only the biomass of gray mullets during winter exhibited a similar trend. During autumn, however, the PDO exhibited the strongest positive correlation with the biomass of gray mullets, followed by the wind speed at the Chang-Yuen Rise.

The annual abundance of mullet since 1912 (Table 3) exhibited the strongest negative correlation with the SST anomaly at the Chang-Yuen Rise, followed by the SST anomaly in the littoral waters of China throughout the year; here, the abundance of gray mullet exhibited comparable trends across all seasons. The strongest positive correlation between wind speed and the annual abundance of gray mullet was identified in the coastal waters of China, followed by the Chang-Yuen Rise, where only the abundance of gray mullets during winter exhibited a similar trend. However, the PDO exhibited the strongest positive correlation with the abundance of gray mullet in autumn, followed by the wind speed at the Chang-Yuen Rise.

#### Wavelet analysis

**Figure 7:**
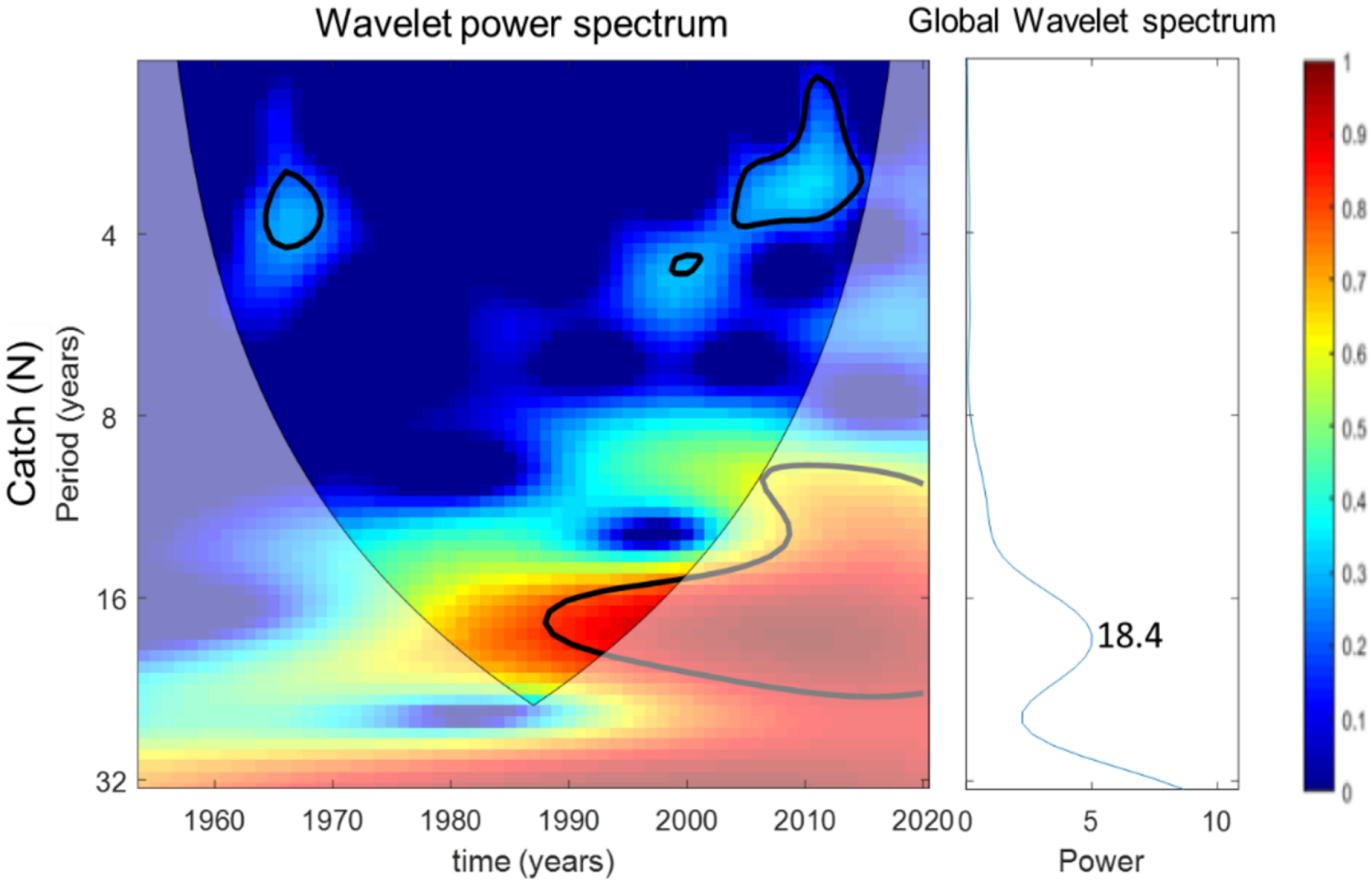
Wavelet analysis results for catch variability over time.

**Figure 8:**
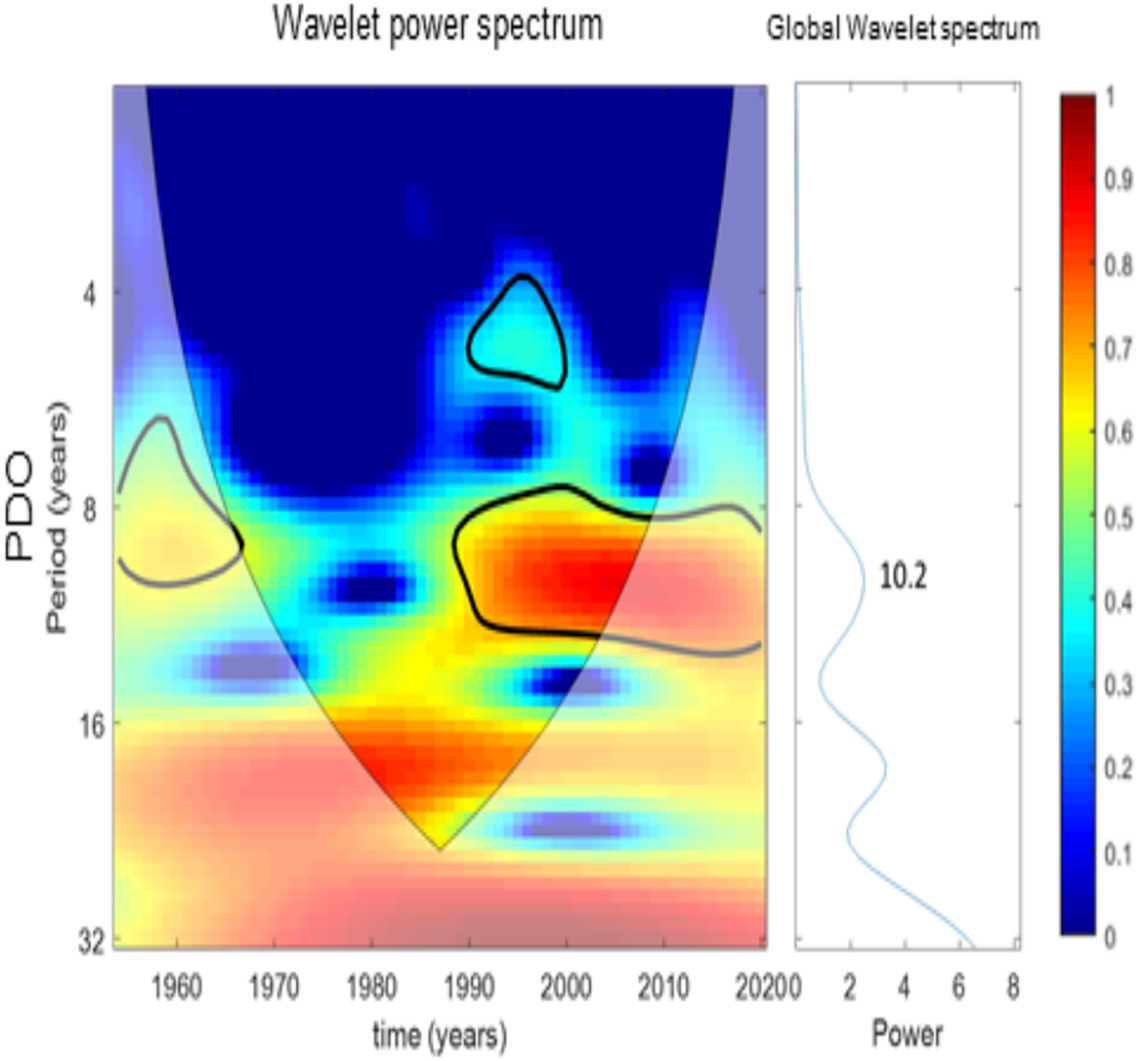
Wavelet analysis results for PDO variability over time.

**Figure 9:**
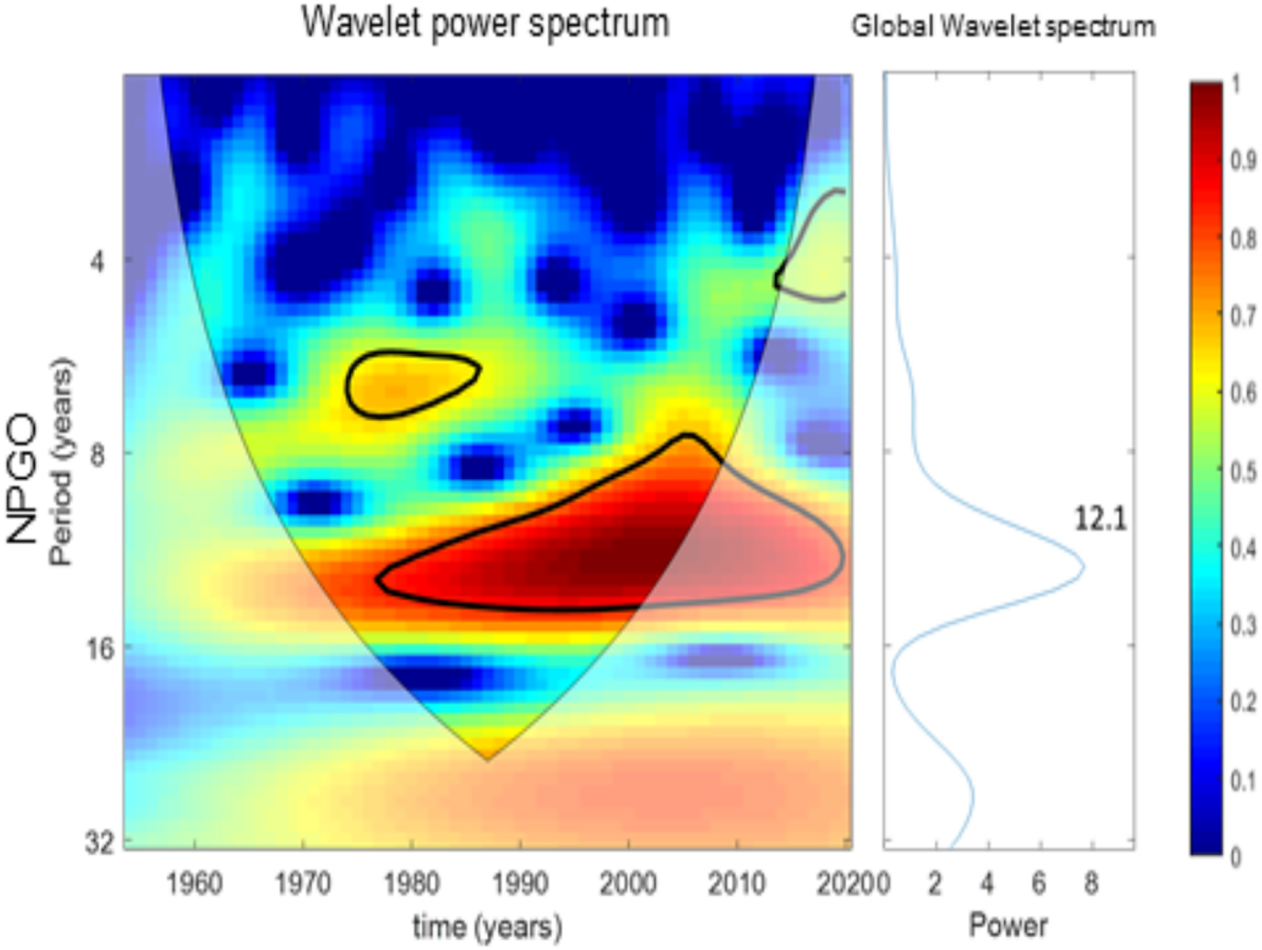
Wavelet analysis results for NPGO variability over time.

**Figure 10:**
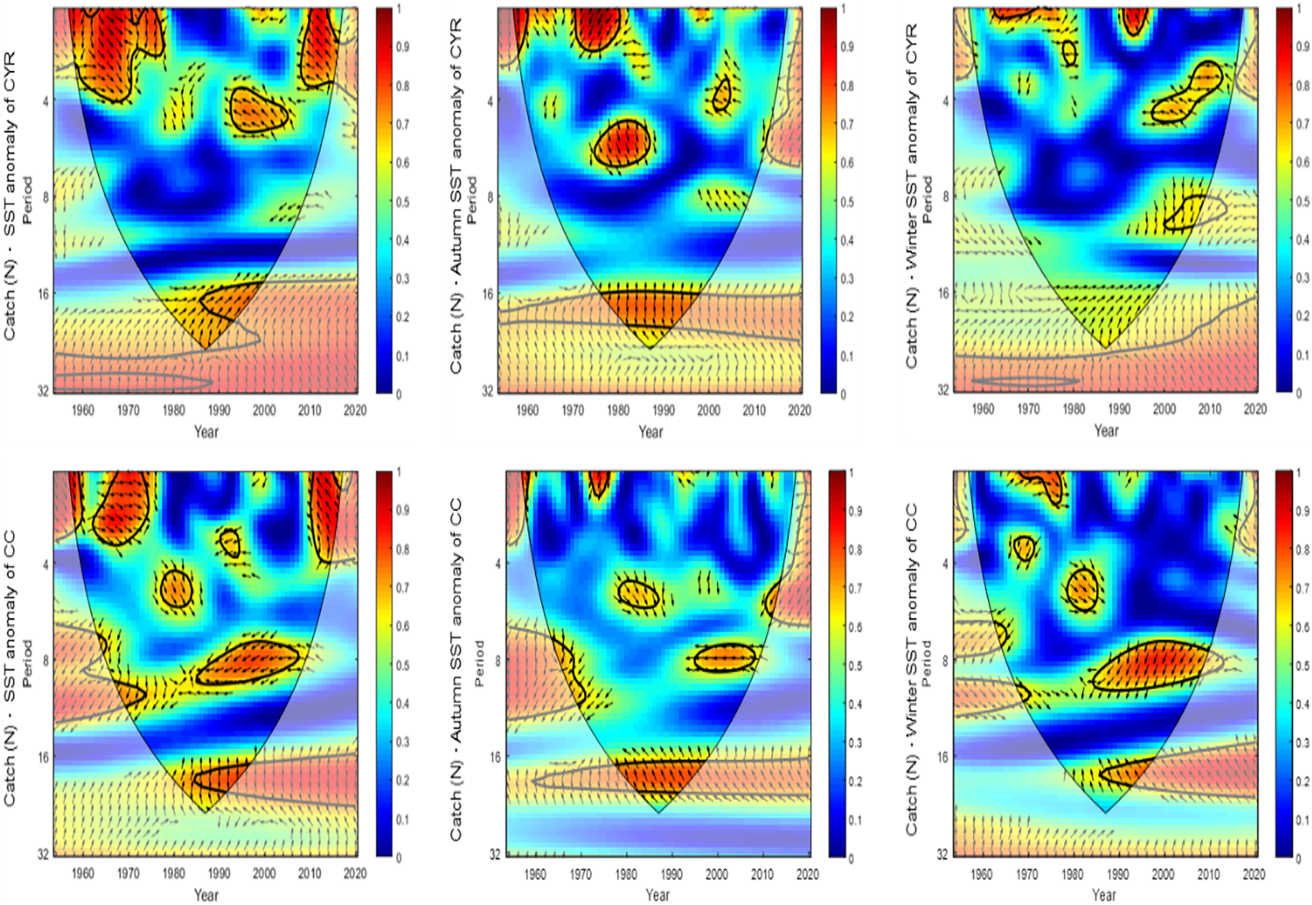
Catch variability with time and SST anomaly variation using wavelet analysis.

Figures 7–10 present the results of wavelet analyses. Figure 7 presents the catch variability over time and reveals that the maximum cyclic period associated with the strongest correlation was 18 years. Figure 8 presents the temporal variability of the PDO and reveals that the maximum cyclic period associated with the strongest correlation was 10 years. Figure 9 presents the variability of the NPGO over time and reveals that the maximum cyclic period associated with the strongest correlation was 12 years.

Figure 10 presents the catch variability over time and the SST anomaly variation at the Chang-Yuen Rise. For the 1970–1980, 2000–2010, and 1970–1980 periods, strong positive correlations between catches and SST anomalies were identified for the 2–4-year, 4–5-year, and 5-year periodic cycles, respectively.

### Stakeholder perceptions of effects of climate change on gray mullet production

The fishermen who participated in our survey asserted that climate change was responsible for the decrease in gray mullet production. In addition, the difficulty of recruiting Taiwanese fishermen and the rapid development of fisheries technology in mainland China have intensified the competition for gray mullet fishing grounds. Furthermore, the price of gray mullet varies across markets, which is disadvantageous to fishermen. The surveyed fishermen believed that local fisheries could not increase their gray mullet production because of climate change, which is a natural phenomenon. The surveyed seafarers expressed strong optimism regarding sustainable management. In addition, the advancement of gray mullet aquaculture has led to the development of enhanced techniques that prevent overfishing and provide target species with adequate time and space to mature and spawn.

According to the Taiwanese government, the present variation in gray mullet production is a result of climate change, which poses a major threat to the fishing industry. The increasing SST and the exploitation and competition associated with mainland Chinese fishing operations may further contribute to a substantial decrease in gray mullet catches. Thus, the surveyed fishermen concluded that aquaculture techniques for cultivating gray mullet must be improved and that the long-term relationship between natural resources and climate change must be investigated. This finding highlights the necessity of developing methods for predicting gray mullet behavior and enhancing fishery management procedures. The fishermen also suggested that negotiations with mainland China fisheries should be conducted to identify appropriate solutions or gray mullet imports from mainland China should be banned. For sustainable management, they suggested that exploitation should be avoided and that the relevant aquaculture technologies and processed products should be developed. In addition, they emphasized the necessity of establishing sanitation inspection standards for mullet roe products and other related products. Policies and collaborations with neighboring countries must also be established to promote and achieve resource sustainability.

According to the researchers surveyed, climate change is the primary factor contributing to the declining trend of gray mullet migration. In addition, the competition from mainland Chinese fishermen and the depletion of aquaculture resources are currently the major challenges for Taiwan’s gray mullet fisheries. Thus, developing intelligent aquaculture solutions and enhancing the current understanding of climate change through continued research are necessary. Furthermore, Taiwan’s ability to manage its natural resources can be enhanced with the support of monitoring techniques, and this can be achieved by establishing partnerships among industry, government, and academic organizations. Additionally, sustainable industrial development should be bolstered.

**Figure 11:**
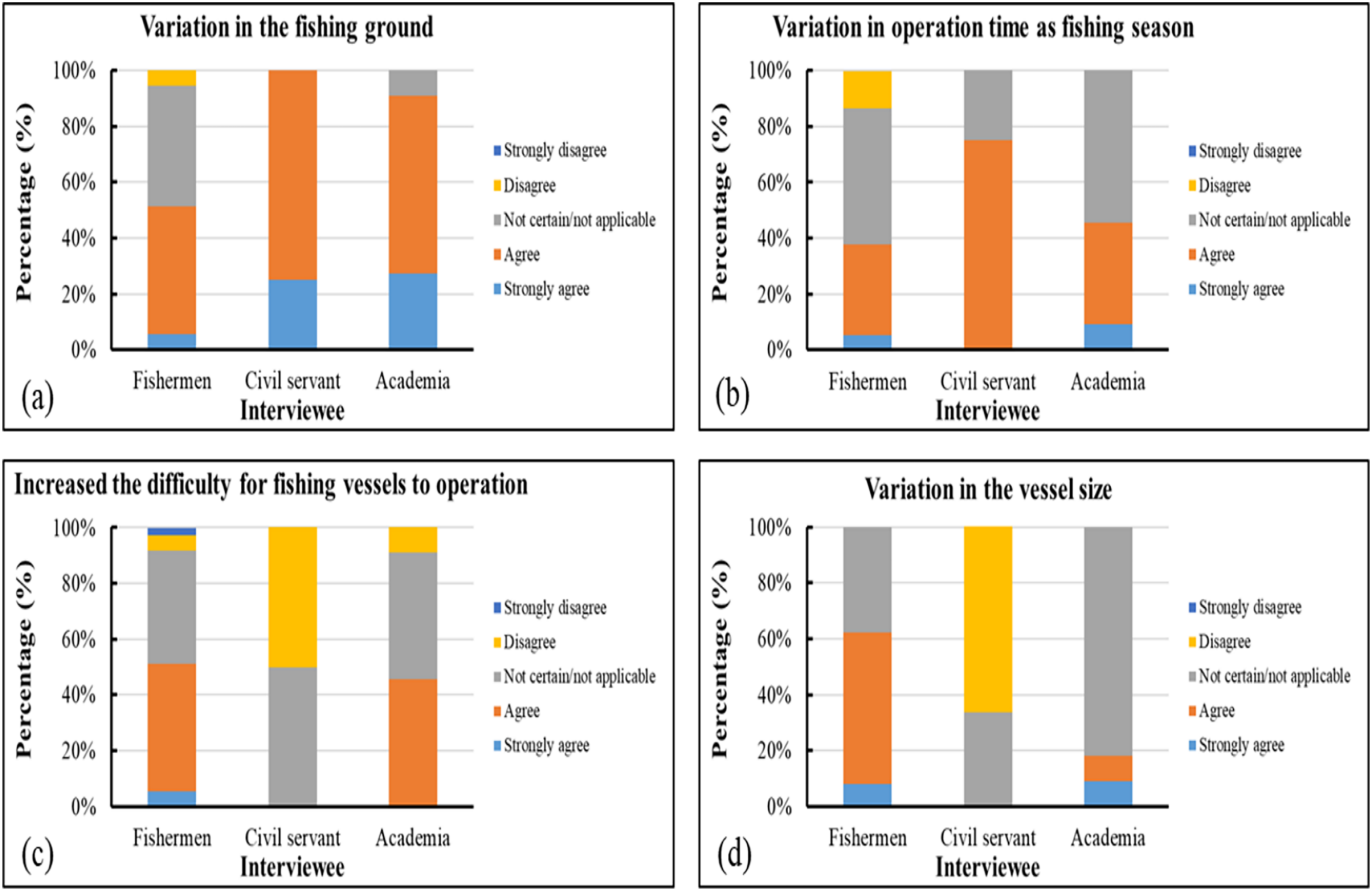
(A–D) Survey results related to fishing operations.

Most of the surveyed government personnel and researchers who responded to our questionnaire believed that variations in fishing grounds affected the production of gray mullet, and nearly half of the surveyed fishermen agreed with this argument. In contrast to other participant groups, most of the government personnel felt that climate change extended the operating season and that climate variability did not increase the difficulties encountered by fisheries. Approximately 60% of the surveyed fishermen reported that the dimensions of fishing vessels changed as a result of climate change (Fig. 11).

**Figure 12:**
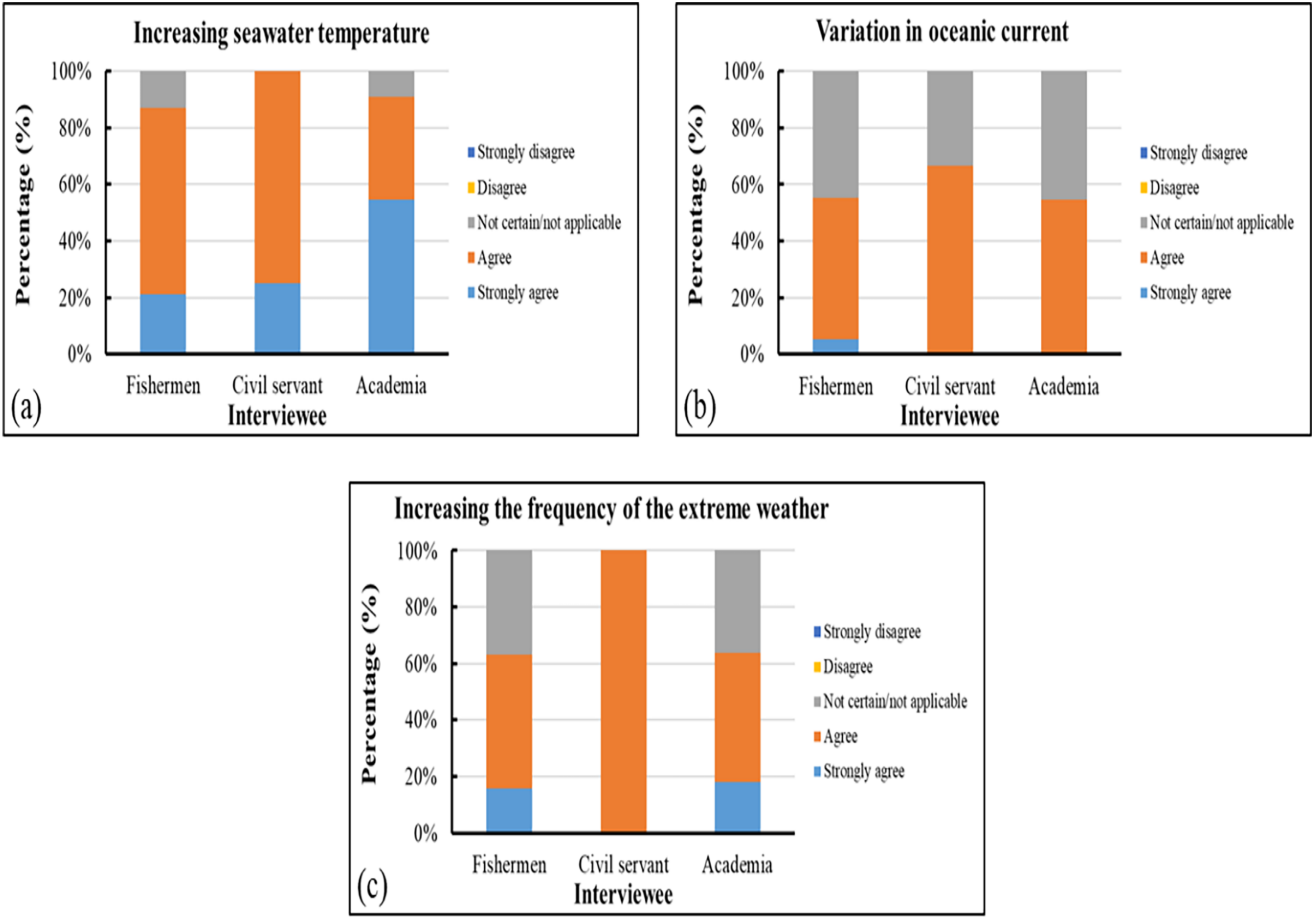
(A–C) Survey results related to fishing conditions.

Nearly all survey participants agreed that changes in SST, oceanic currents, and the frequency of extreme weather events affected the gray mullet fisheries in the study area. In addition, 33–46% of the participants expressed uncertainty about the effects of changes in oceanic currents, with approximately 37% of the surveyed fishermen and nearly 36% of the surveyed academics asserting that the increasing frequency of adverse weather events was unrelated to the changes in gray mullet production (Fig. 12).

**Figure 13:**
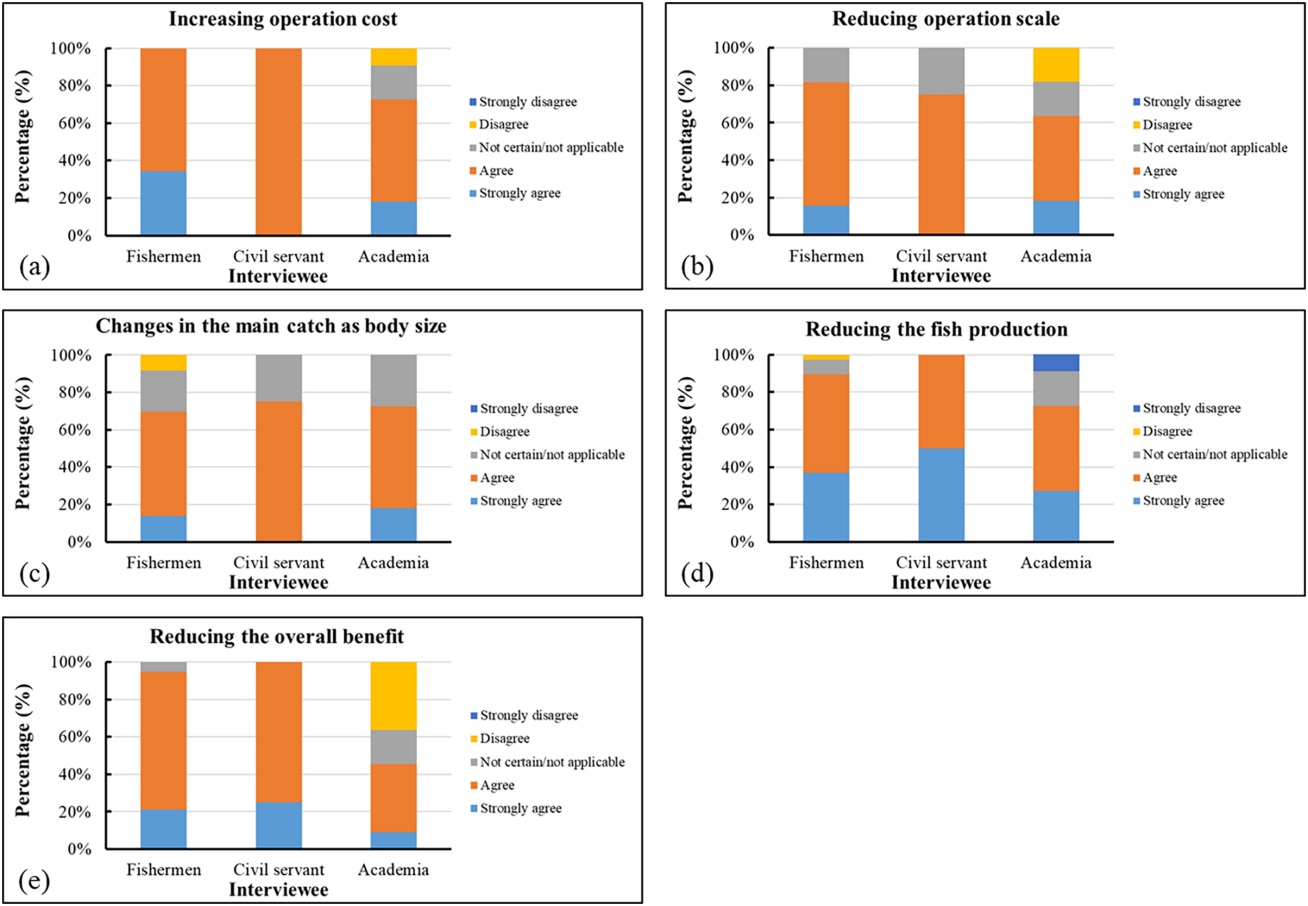
(A–E) Survey results related to economic conditions.

Most of the surveyed participants concurred that climate change has led to increased operational costs and a decrease in available fishing grounds and production areas. For variations in primary catches, more than 90% of the surveyed participants concurred that climate change was the main factor influencing changes in the body size of caught gray mullets. Therefore, although approximately 36% of the experts disagreed with this assertion, climate variability could still be a key cause of the decline in overall catch (Fig. 13).

## Discussion

### Role of gray mullet fisheries in Taiwan

In Taiwan, the gray mullet is a major species in both aquaculture and small-scale fishing, and it has long been a primary target of traditional fisheries. In addition, processed gray mullet products have a high market value because the roe, testes, and stomach of the gray mullet are regarded as delicacies. Therefore, a balance must be struck between the use and protection of gray mullets. For instance, the production of roe by gray mullets is a process that requires 10–14 days and comprises nine phases. Notably, after the third phase, the ovaries of cultured gray mullets, which contain a high amount of oil, are preserved for 1 week. Finally, the expansion of the gray mullet industry in Taiwan is dependent on maintaining a balance between capture fisheries and aquaculture, making technological advances, and developing novel methods for marketing gray mullet.

### Variability in gray mullet catches

Prior to 1990, the CCC would always pass over the Yun Chang Rise approximately 20 days before the winter solstice and form a low-temperature, low-salinity, and frigid water tongue off the southwestern coast of Taiwan; this phenomenon occurred annually unless a given year was a La Niña year. The gray mullets would then deposit their eggs in this area, which is 30–50-m deep and dominated by steep underwater terrain, making it an excellent location for gray mullet larvae to feed because of the northeast monsoon and offshore upwelling. However, the present study revealed that the Kuroshio Current strengthened after 1998 and during the La Niña era. Since 1998, the Kuroshio Current and CCC have converged primarily north of the Yun Chang Rise. High waves prevent gray mullets from laying eggs near the coastlines of Taoyuan, Hsinchu, and Miaoli. Because gray mullets cannot travel further south, they follow the CCC to the northeastern coast of Taiwan (*i.e*., Yilan) to spawn, where they are then caught by fishermen. However, further research is required to determine whether this area is suitable for larval development. Prior to 1990, gray mullets could spawn over the Yun Chang Rise because this rise obstructed wind and waves, and they could be caught using only purse seines. Since 1990, however, gray mullets have been primarily caught using gill nets off the coastlines of Taoyuan, Hsinchu, and Miaoli. Although purse seines were used off the coasts of Keelung and Yilan, the catches were limited.

Two topics that require further clarification are whether the gray mullets migrating from China were caught by Chinese fishermen or whether these gray mullets have sought out new spawning grounds. The changes in the annual catch of gray mullet in Taiwan indicate that climate change affects the marine ecosystem and fisheries. Although several factors that influence the depletion of natural resource and climate change have not yet been identified, this topic must be studied in accordance with the maxim of thinking globally and acting locally.

Increases in SST could have been due largely to the decline of gray mullet catches from 1980 onward. From 1984 to 2009, the average SST at Chang-Yuen Rise increased by 1.01 °C. Between 1958 and 1998, the winter 20 °C isotherm in Taiwan shifted from 23°N–24°N to north of 25°N. After the changes that occurred to the 20 °C isotherm in Taiwan, the sites for fishing gray mullet also shifted northward. In conclusion, climate change scenarios created using the IPCC A2 model predicted that the 20 °C isotherm in the Taiwan Strait would shift to 25.5°N by 2050 and cross 26°N by 2075. Consequently, rippling fishing may become feasible in Taiwan and the rest of the world in the near future. Changes in catch levels (fluctuations), climate indices, and costs encouraged the use of purse-seiner and gillnet vessels (Lan et al., 2014). Since 2013, the most prevalent fishing technique has involved the use of gill nets because of their affordability and the low fuel requirement of this technique (Neumann, Ott & Kenchington, 2017). The present results can be used to interpret the effects of fisheries resources on the environment, habitat, and community structure; they can also serve as a reference for developing management recommendations that correspond to SDGs 2, 8, 13, and 14 (Ntona & Morgera, 2018). Sustainable development requires the consistent collection of sample fishery data, the classification of gray mullets by length range, and the establishment of an HSI model for the various stages of the gray mullet’s life cycle (Shao et al., 2011).

Shen et al. (2011) identified three cryptic *M. cephalus* species in the northwestern Pacific Ocean and suggested that the distributions of the three species mostly corresponded to the three major oceanographic current systems, namely the South China Current, CCC, and Kuroshio Current. However, the recent changes and decreases in gray mullet populations may be due to the following three reasons:

1. The preferred environment of the gray mullet in the study region could have been disrupted by climate change (Brusle, 1981).
2. The continuous effect of the El Niño, La Niña, and El Niño–Southern Oscillation (ENSO) phenomena could have reduced the catch of gray mullet.
3. Efforts to fish for this fish species could have been inhibited by SDG-related government policies.

Additionally, gray mullets could have changed their migrating behavior to adapt to the occurrences of El Niño and La Niña events. We generally observed that gray mullets preferred a pelagic environment during La Niña events but migrated toward a demersal environment during El Niño events. From 2002 onward, the continual occurrence of El Niño events (2002–2003, 2004–2005, 2006–2007, 2009–2010, 2014–2016, and 2018–2019 periods) followed by La Niña events (2000–2001, 2008–2009, 2010–2011, and 2016–2017 periods) was strongly correlated with gray mullet migration (Ibáñez et al., 2012). Furthermore, the key factors that contributed to the present condition of gray mullets comprise the following:

1. The fishing fleets from mainland China that target the gray mullet have expanded in size (Hung & Shaw, 2006).
2. Overfishing in the study area could have contributed to the decrease in the annual catch of gray mullets in the 1980s (Tung, 1981, pp. 17–18).

Climate change and global warming–related increases in SST are also crucial factors that could have reduced the gray mullet catch in Taiwan (Lan et al., 2014).

### Management strategy recommendations based on questionnaire survey results

According to the surveyed fishermen, the decrease in gray mullet production occurred because of climate change. Recruiting Taiwanese fishermen is a challenging task (González-Castro, Macchi & Cousseau, 2011; Hotos & Vlahos, 1998). Furthermore, fisheries technology in mainland China has advanced swiftly, which has intensified the competition for gray mullet fishing grounds. The cost of producing gray mullet varies considerably among markets, and this can have a negative effect on the earnings of fishermen. The surveyed fishermen also asserted that the production of gray mullet could not be increased after the occurrence of climate change because climate change is a natural phenomenon. Fishermen have expressed limited confidence in sustainable management (Chen, 2006). They have also asserted that the overexploitation of gray mullet must be prevented, gray mullets must be allowed to mature and reproduce, gray mullet aquaculture must be improved, and more effective fishing techniques must be developed and implemented.

Taiwan’s government stated that climate change is the cause of the observed fluctuations in gray mullet production. In addition to overfishing and competition from mainland Chinese fishermen, an increasing SST has also contributed to the rapid decrease in gray mullet catch rates. However, further investigation should be conducted to clarify the long-term relationship between natural resources and climate change and to advance aquaculture techniques for cultivating gray mullet (Crosetti, 2015). A prediction system for this species should also be developed to enhance fisheries management that focuses on gray mullet production. Negotiations with Chinese mainland fishermen should be conducted to pursue viable solutions or the import of Chinese mainland mullet products could be banned. From the perspective of sustainable management, preventing exploitation and developing the appropriate aquaculture technologies and processed products are essential tasks. In addition, collaborations with neighboring countries should be established to develop and implement sound rules for fishery operations, and inspection criteria for gray mullet roe and related products should be developed and applied (Chien, Kirby & Sheen, 2016). According to the surveyed specialists, climate change is the primary reason for the decline in gray mullet migration. Moreover, the competition with mainland Chinese fishermen and the current condition of Taiwan’s aquaculture industry pose major challenges for Taiwan’s gray mullet fisheries. To develop intelligent aquaculture and expand our understanding of climate change, the aforementioned topics should be further explored. The researchers of the present study suggest that relationships among business, government, and academic organizations should be established or strengthened to enhance the monitoring and management of natural resources. Furthermore, promoting sustainable industrial development is another goal that must be pursued.

Our survey results indicated that most of the surveyed government employees and academics were of the opinion that the changes in gray mullet fishing grounds influenced the yield of this target species; by contrast, only approximately half of the surveyed fishermen shared this opinion. In addition, most of the surveyed government employees believed that climate change prolonged the suspension of fishery operations and that climatic variability increased the difficulty of conducting fishing activities. Approximately 60% of the surveyed mariners believed that the changes in the sizes of fishing vessels were caused by climate change. Most of the surveyed participants agreed that various variations in ocean currents, extreme weather, and SST influenced the operations of gray mullet fisheries in the study area. Approximately 37% of the surveyed fishermen and nearly 36% of the surveyed academics believed that the increasing frequency of extreme weather was unrelated to the changes in the production of gray mullet. Most of the participants agreed that the economic effects of climate change included an increase in operating expenses and reductions in available fishing grounds and fish yields. For primary catch variation, >90% of the surveyed participants agreed that climate change was the most crucial factor influencing the changes in the body size of caught gray mullets, primarily through exploitation and size selectivity (Palkovacs, 2011; Liang et al., 2014). Consequently, although approximately 36% of the surveyed experts disagreed with this assertion, climate variability could still be a key cause of the decrease in yield.

The production of marine fish is highly dependent on ocean conditions. Temperature fluctuations can influence how fish function and their choice of habitat (Genner et al., 2010; Gittings et al., 2018; Perry et al., 2005). When a species migrates from one location to another, its population structure is altered, affecting how fishing is conducted, how catch portions are distributed and how the effectiveness of fishery management is evaluated (Gittings et al., 2018). Accordingly, climate change can affect the quantity, composition, and distribution of fisheries resources, which can in turn increase the cost of fishing vessels and other fishing equipment (Pauly, Watson & Alder, 2005). According to Rudea et al. (2011), the decrease in fish supply and increase in fish prices may be sufficient to offset the decrease in gross revenue. According to Lam et al. (2016), a shift in fishing grounds increases the cost of deep-sea fishing operations, which is detrimental to global fisheries and expected to reduce their future revenue by 35%. Additionally, long-term changes in ocean conditions cause fishing grounds to shift, increasing the cost of operations for fisheries and forcing them to consume more fuel to perform fishery operations (McIlgorm et al., 2010). Consumer surplus decreases when prices increase (Sumaila et al., 2011), and additional indirect costs may also be incurred (*e.g*., boat construction or maintenance costs, equipment supply costs, and business sector costs; Lam et al., 2016). Genner et al. (2010) demonstrated that the variations in a thermal regime can affect the abundance and sizes of various fish species, revealed a fundamental long-term variability in their distribution due to northward biogeographic shifts (Perry et al., 2005). The present study focused on the reduction of the primary catch. Gray mullets primarily prey on diatoms (Rudea et al., 2011), which are prevalent in low-temperature, high-nutrient water masses (Zhong et al., 2019). Global warming may reduce the abundance of diatoms (Gittings et al., 2018), and because of the role of prey accessibility in determining body size, this reduction could have resulted in a decrease in the body size of gray mullets, as was hypothesized by a survey participant. Several studies have also suggested that the competition for gray mullet fishing grounds between mainland China and Taiwan and gray mullet overexploitation contributed to the decrease in gray mullet production after the 1980s (Huang, Lin & Huang, 2005; Hung & Shaw, 2006; Lan et al., 2017).

### Relevance of SDGs to gray mullet fisheries

SDG 14 was established to facilitate the creation of a sustainable future for our oceans (Urbina & Glover, 2015; Boeuf & Payan, 2001; Rushall, 2007; Kim et al., 2015; Naimullah et al., 2020a, 2020b, 2022; Shiah et al., 2000; Chung, Jan & Liu, 2001). On the basis of the questionnaire survey results, we estimated the effects of climate change on local gray mullet fisheries by conducted Pearson correlation and wavelet transformation analyses. The present study considered exclusive economic zones and fishing grounds from the perspective of SDG 14 (Sturesson, Weitz & Persson, 2018), and it was conducted to facilitate the effective management and sustainable use of gray mullet in the study area.

The present study discussed the relevance of the habitats and zones of gray mullets and the potential effects of climate change on their habitats. It also provided recommendations for conserving the habitats of the gray mullet species given the potential effects of climate change. Overfishing and other harmful fishing methods, the expansion of hypoxic or “dead” zones at sea, the warming and acidification of the ocean, and the spread of invasive species are the key threats to ocean resources today. Additionally, the introduction of new, high-cost methods for acquiring resources may cause less technologically advanced countries to fall behind their competitors in the use of limited marine resources, thereby increasing global inequality. Ocean ecosystems can be conserved by changing or substantially reducing their use, and this step is necessary given the exacerbation of climate change and the major role of the processes that cause ocean warming, rising sea levels, and acidification in changing the lives and livelihoods of billions of people worldwide (Virto, 2018).

## Conclusion

Our findings revealed a downward trend in the annual catch of gray mullet since the 1980s, notably between 1986 and 2010. We analyzed the dynamic changes in fishing environments resulting from climate change, identifying the northward shift of fishing grounds in response to changes in the 20 °C isotherm in Taiwan’s coastal waters. The migration of flathead gray mullets was discovered to be strongly correlated with SST, the ENSO, and the PDO but not with climate-related factors (*e.g*., daily atmospheric temperature, atmospheric pressure, and wind speed and direction). The results of our Pearson correlation analysis and wavelet analysis indicated that the decrease in the gray mullet population was a complex process that could be attributed to several main factors, which are discussed in the present study. Thus, exploitation must be examined in the context of the migration of gray mullets, which was primarily caused by climate change and habitat transformation. In addition, a survey of stakeholders furthered our understanding of the behavioral pattern of gray mullets. Understanding the changes in fish population dynamics caused by climate change and their repercussions can facilitate the development of robust policies that promote the sustainable growth of Taiwan’s fishery industry.

## Supplemental Information

10.7717/peerj.15788/supp-1 Supplemental Information 1 Questionnaire.

10.7717/peerj.15788/supp-2 Supplemental Information 2 Raw Data.

## Additional Information and Declarations

### Data Availability

The following information was supplied regarding data availability: The raw data is available in the Supplemental File.
