# Supplementary material for: Fishery-based adaption to climate change: the case of migratory species flathead grey mullet (Mugil cephalus L.) in Taiwan Strait, Northwestern Pacific
Source: PeerJ. 2023 Aug 30;11:e15788. doi: 10.7717/peerj.15788 (PMC10474836; doi:10.7717/peerj.15788)
Supplement: Supplemental Information 1 [file peerj-11-15788-s001.docx]

**QUESTIONNAIRE SURVEY FOR THE IMPACTS OF CLIMATE CHANGE ON THE GREY MULLET FISHERY**

**Part 1:** Basic information

1. Name:___________________ Gender: □Male □Female Tel: ______________
2. Age: □Under 30 □31~40 □41~50 □51~60 □60 or above
3. Occupation: □Fisherman □Other (please go to part 2): _______________
4. Engage in the grey mullet fishery: □No (please go to part 2) □Yes, from______to_______
5. What kind of fishing gear:

□Purse seine □Trawl □Gillnet □Other: ________________

1. The tonnage of vessel:

□Unpowered sampan (CTX) □Unpowered fishing raft (CTY)

□Powered sampan (CTS) □Powered fishing raft (CTR)

□Under 10 tons (CT0-1) □10-20 tons (CT2)

□20-50 tons (CT3) □50 tons or higher (CT4 or higher)

1. The monthly income:

□Under 20,000NTD □20,000-40,000NTD □40,000-60,000NTD

□60,000-80,000NTD □80,000-100,000NTD □higher than 100,000NTD

**Part 2:** The impacts of climate change on the grey mullet fishery

1. Based on your experience, how has the grey mullet fishery been varied by the impacts of climate variability:

|  | Strongly disagree | Disagree | Not certain/ not applicable | Agree | Strongly agree |
| --- | --- | --- | --- | --- | --- |
| a. Economic aspect | | | | | |
| Increasing operation cost |  |  |  |  |  |
| Reducing operation scale |  |  |  |  |  |
| Changes in the main catch as body size |  |  |  |  |  |
| Reducing the fish production |  |  |  |  |  |
| Reducing the overall benefit |  |  |  |  |  |
| b. Environmental aspect | | | | | |
| Increasing seawater temperature |  |  |  |  |  |
| Variation in oceanic current |  |  |  |  |  |
| Increasing the frequency of the extreme weather |  |  |  |  |  |
| c. Operation aspect | | | | | |
| Variation in the fishing ground |  |  |  |  |  |
| Variation in operation time as fishing season |  |  |  |  |  |
| Increasing the difficulty for fishing vessel to operation |  |  |  |  |  |
| Variation in the vessel size |  |  |  |  |  |

1. In your opinion, what are the most important issues in the current Taiwanese grey mullet fishery?

________________________________________________________________________________

________________________________________________________________________________

________________________________________________________________________________

1. For the aforementioned issues in the grey mullet industry, do you have any solution or suggestion?

________________________________________________________________________________

________________________________________________________________________________

________________________________________________________________________________

1. What do you think about sustainable development for grey mullet fisheries?

________________________________________________________________________________

________________________________________________________________________________

________________________________________________________________________________
